# Long-term efficacy and safety of adalimumab in 4-12 year old patients with juvenile idiopathic arthritis

**DOI:** 10.1186/1546-0096-9-S1-O26

**Published:** 2011-09-14

**Authors:** N Ruperto, DJ Lovell, A Reiff, M Gamir, G Higgins, I Koné-Paut, OY Jones, E Chalom, N Ilowite, C Wouters, MJ McIlraith, S Liu, H Kupper, EH Giannini, A Martini

**Affiliations:** 1 PRINTO-IRCCS Genova, Italy; 2 PRCSG-Cincinnati Children’s Hospital Medical Center, Cincinnati, USA; 3 Abbott, Rungis, France; 4 Abbott, Abbott Park, USA; 5 Abbott, Ludwigshafen, Germany

## Background

Adalimumab (ADA) has been shown to be safe and effective in a study of juvenile idiopathic arthritis (JIA) patients aged 4-17 years at a dose of 24 mg/m^2^ every other week.^1^

## Aim

To determine the long-term efficacy and safety of ADA in a subgroup of BSA-dosed JIA patients aged 4-12 years.

## Methods

Patients with polyarticular course JIA (N=171) were enrolled in a phase 3, randomized-withdrawal, double-blind (DB), stratified, parallel-group study, which consisted of a 16-wk open-label (OL) lead-in, a 32-wk DB phase, and an up to 5 year OL extension phase that included BSA dosing prior to a switch to fixed dose (FD). Patients on ADA (24 mg/m^2^; max dose, 40 mg) were evaluated based on ACR pediatric response criteria for improvement (ACR Pedi 30/50/70/90) and monitored for adverse events (AEs).

## Results

Subjects in the 4-12-year-old subgroup (n=41) were 73% female and 98% white, with a mean age of 9 years. At baseline, mean PhyGA was 58.1, mean PaGA was 47.9, AJC was 18.6, and DI-CHAQ was 1.0. ACR Pedi 30/50/70/90 responses at wk 106 (Table) were comparable to completer patients (n=62) in the overall population who reached ~240 wks in the OL extension FD phase (95%/90%/82%/69%). Improvements in core disease activity/severity variables were maintained throughout the study (Table 1). The most common AEs were infections and injection site reactions; there were no opportunistic infections, malignancies, or deaths. One patient in the 4-12-year-old subgroup discontinued due to AEs (viral illness and hydrocephalus secondary to mechanical complications of nervous system device).

**Table 1:** ACR indicates American College of Rheumatology; AJC, active joint count; DI-CHAQ, disability index of childhood health assessment questionnaire; PaGA, parent global assessment of well-being; PhyGA, physician’s global assessment of disease activity.

Time points	ACR 30/50/70/90	PhyGA	PaGA	AJC	DI-CHAQ
Week 16	**100/88/63/39**	**12.7**	**12.2**	**5.1**	**0.4**

Week 48	**100/96/92/69**	**6.3**	**8.3**	**2.9**	**0.2**

Week 106	**100/96/96/71**	**5.9**	**5.0**	**1.2**	**0.1**

% of Pts Aged 4-12 Years Achieving ACR Pedi and Mean Values in Disease Activity/Severity Variables by ADA Exposure Throughout the Study

## Conclusions

ADA was efficacious and well tolerated in a BSA-dosed JIA subpopulation aged 4-12 years. ACR Pedi 30/50/70/90 responses were maintained through 106 weeks. Overall, efficacy was comparable to the entire study population, in whom maintenance of response was observed throughout the entire study with ADA for up to 6 years.
